# Acid-Labile Surfactants Based on Poly(ethylene glycol), Carbon Dioxide and Propylene Oxide: Miniemulsion Polymerization and Degradation Studies

**DOI:** 10.3390/polym9090422

**Published:** 2017-09-06

**Authors:** Markus Scharfenberg, Sarah Wald, Frederik R. Wurm, Holger Frey

**Affiliations:** 1 Institute of Organic Chemistry, Duesbergweg 10-14, Johannes Gutenberg University Mainz, 55128 Mainz, Germany; mscharfe@uni-mainz.de; 2 Max Planck Institute for Polymer Research, Ackermannweg 10, 55128 Mainz, Germany; wald@mpip-mainz.mpg.de (S.W.); wurm@mpip-mainz.mpg.de (F.R.W.)

**Keywords:** polycarbonate, CO_2_, surfactant, miniemulsion polymerization, degradation, nanoparticle

## Abstract

Partially degradable, nonionic AB and ABA type di- and triblock copolymers based on poly(propylene carbonate) and poly(ethylene glycol) blocks were synthesized via immortal copolymerization of carbon dioxide and propylene oxide, using mPEG or PEG as a macroinitiator, and (*R*,*R*)-(salcy)-CoOBzF_5_ as a catalyst in a solvent-free one-pot procedure. The amphiphilic surfactants were prepared with molecular weights (*M_n_*) between 2800 and 10,000 g·mol^−1^ with narrow molecular weight distributions (1.03–1.09). The copolymers were characterized using ^1^H-, ^13^C- and DOSY-NMR spectroscopy and size exclusion chromatography (SEC). Surface-active properties were determined by surface tension measurements (critical micelle concentration, CMC; CMC range: 1–14 mg·mL^−1^). Degradation of the acid-labile polycarbonate blocks was investigated in aqueous solution using online ^1^H-NMR spectroscopy and SEC. The amphiphilic polymers were used as surfactants in a direct miniemulsion polymerization for poly(styrene) (PS) nanoparticles with mean diameter of 270 to 940 nm. The usage of an acid-triggered precipitation of the emulsion simplified the separation of the particles from the surfactant and purification of the nanoparticles.

## 1. Introduction

Surfactants are used for a broad scope of applications. They are employed in the textile, paint, and oil industry, in cleaning agents and cosmetics, for microelectronics and optics, and further possible applications are found in biomedicine, pharmaceutical products and nanoscience [1,2,3,4]. Another important field of application of surfactants is emulsion polymerization, which plays a key role in many industrial areas. Miniemulsion polymerization is a special case, where nanodroplets are stabilized by a rather low amount of surfactant against coalescence and additionally against Ostwald ripening, using an osmotic agent. With the help of this heterophase technique structured polymeric particles can be obtained and different solid or liquid cargo molecules can be encapsulated [5]. Removal of the surfactant from the formed nanoparticles might be desirable for several applications, since a large amount of surfactant can be problematic, because of cytotoxicity or interfere with film forming properties of the dispersion [6,7]. In this context a promising strategy is the degradation of the surfactant or switching its amphiphilicity by an external trigger, e.g., pH variation or redox conditions directly in the dispersion [8,9,10]. Aliphatic polycarbonates are degradable and therefore promising materials for degradable surfactants. Furthermore, they can be synthesized via direct copolymerization of epoxides and carbon dioxide.

Carbon dioxide is a renewable, sustainable, non-toxic and non-flammable C_1_ feedstock that can be obtained inexpensively in large quantities. Its use is limited because of its thermodynamic stability and low reactivity [11,12]. Since the development of the immortal CO_2_/epoxide polymerization by Inoue et al. in 1969, carbon dioxide has also been used as a feedstock for polymers. In combination with epoxides aliphatic polycarbonates are obtained [13,14]. Mostly propylene oxide (PO) and cyclohexene oxide (CHO) have been copolymerized with CO_2_ to date, resulting in biodegradable materials [15,16].

In the literature several degradation studies of aliphatic polycarbonates were published focusing on acidic, basic and enzymatic conditions. However, none of the authors investigated the degradation of polycarbonates dissolved in an aqueous solution because of the insolubility and apolar character of common aliphatic polycarbonates [17,18,19,20,21,22]. In the respective works polycarbonates with special functional groups (e.g., hydroxyl or indene groups) were analyzed with respect to their depolymerization in organic solvents [23,24,25]. Water-soluble aliphatic polycarbonates have been described. However, their degradation behavior was not investigated [26,27,28,29,30]. In a comprehensive work of Hauenstein et al. limonene based, water-soluble polycarbonates were investigated regarding their degradation in aqueous solution at different pH values. The lack of degradation observed in this work was explained by steric shielding due to the side chains [31]. Darensbourg and coworkers focused on the degradation of water-soluble polycarbonates in basic conditions using deprotonated carboxylic acid pendent groups as side chains [32]. Reports on water-soluble block copolymers based on polycarbonates are limited and to the best of our knowledge, none of them was ever used as a surfactant [26,27,28].

In the current work, the use of amphiphilic polycarbonate block copolymers as nonionic, acid-labile surfactants for an oil-in-water miniemulsion is presented. The amphiphilic structures are prepared from hydrophilic poly(ethylene glycol) (PEG or mPEG) acting as a transfer agent, i.e., as an initiator [27,33], and a hydrophobic poly(propylene carbonate) (PPCs), which was prepared directly from carbon dioxide and propylene oxide. The surfactants show excellent solubility in water and aggregate spontaneously to form micelles in an aqueous solution. Furthermore, we investigate the degradation of the surfactants via hydrolysis and their depolymerization in water under acidic conditions. Finally, separation of the surfactant and the PS nanoparticles was studied.

## 2. Experimental Section

### 2.1. Materials

All solvents and reagents were used as received, except it is described otherwise. Propylene oxide (PO, 98%, Sigma Aldrich, St. Louis, MO, USA) was distilled over CaH_2_ under reduced pressure prior to use. Carbon dioxide (>99.99%) was purchased from Westfalen AG (Münster, Germany). Lutensol AT50 was purchased from BASF (Ludwigshafen, Germany). All solvents were purchased from Sigma Aldrich, Fisher Chemical (Pittsburgh, PA, USA) and Honeywell (Morris Plains, NJ, USA). All reagents were purchased from Sigma Aldrich or Acros Organics (Pittsburgh, PA, USA). Deuterated solvents were received from Deutero GmbH (Kastellaun, Germany).

### 2.2. Measurements

^1^H- and ^13^C-NMR spectra were recorded on a Bruker 400 spectrometer (Bruker Corporation, Billerica, MA, USA), operated at 400 MHz and 100 MHz, respectively, at 21 °C and the chemical shifts are given in parts per million (ppm). All spectra are referenced to residual solvent signal. For size exclusion chromatography (SEC) measurements in DMF (containing 0.25 g·L^−1^ of lithium bromide as an additive) an Agilent 1100 Series system (Agilent Technologies, Santa Clara, CA, USA) was used as an integrated instrument, including a PSS HEMA column (Mainz, Germany, 10^6^/10^5^/10^4^ g·mol^−1^), a RI and an UV detector. Calibration was carried out using poly(ethylene glycol) standards provided by Polymer Standards Service (Mainz, Germany). FT-IR spectra were recorded using a iS10 FT-IR spectrometer (Thermo Scientific, Waltham, MA, USA) equipped with a diamond ATR unit. Surface tension measurements to determine the critical micelle concentration were performed using a DCAT 11 EC tensiometer (Dataphysics, Filderstadt, Germany) equipped with a TV 70 temperature control unit, a LDU 1/1 liquid dosing and a refill unit, as well as a RG 11 Du Nouy ring. Surface tension data were processed using SCAT v3.3.2.93 software. All solutions were stirred for 120 s at a stirring rate of 50%. The tension values were measured three times after 300 s. The CMC was determined by linear regression of the slopes at high and at low concentration. The point of intersection was the CMC. The Du Nouy ring was washed with water and annealed in a butane flame prior to use. The nanoparticle formation was detected by scanning electron microscopy (SEM) using a 1530 LEO Gemini microscope (Zeiss, Oberkochen, Germany). The nanoparticle dispersion (10 µL) was diluted in 3 mL distilled water, drop-cast onto silica wafers, and dried under ambient conditions. Afterwards the silica wafers were placed under the microscope and each sample was analyzed at a working distance of ~3 mm and an accelerating voltage of 0.2 kV. Dynamic light scattering (DLS) was used to determine the hydrodynamic diameter of the generated nanoparticles in water at a Nicomp 380 Submicron particle Sizer (PSS-Nicomp, Port Richey, FL, USA) at a fixed scattering angle of 90°. 10 µL of the emulsion was diluted in 3 mL distilled water.

### 2.3. Synthesis of (R,R)-(salcy)-CoOBzF_5_

(*R*,*R*)-(salcy)-CoOBzF_5_ was prepared as described by Coates et al. [34].

### 2.4. General procedure for the Synthesis of mPEG-b-PPC and PPC-b-PEG-b-PPC

mPEG was dried by azeotropic distillation using benzene under reduced pressure. A 100 mL Roth autoclave was dried under vacuum at 40 °C for 24 h. mPEG_113_ (3 g, 0.6 mmol), propylene oxide (2.6 mL, 37.2 mmol), (*R*,*R*)-(salcy)-CoOBzF_5_ (13.1 mg, 0.016 mmol) and [PPN]Cl (8.7 mg, 0.015 mmol) were combined with a stir bar inside the autoclave under inert gas atmosphere. The mixture was stirred under pressure of 50 bar CO_2_ at 30 °C for 23 h. The crude product was dissolved in 10 mL CH_3_CN and quenched with 6 mL 5% HCl solution in methanol. The solution was precipitated in cold diethyl ether, the precipitate collected by centrifugation and redissolved in 10 mL THF. A column with neutral aluminum oxide was used for complete removal of the catalyst using THF as an eluent. Fast work up is crucial when using aluminum oxide, because of the fast degradation of the polycarbonate block in the presence of aluminum oxide. The product changed its color from green to colorless and was precipitated in cold diethyl ether once more. The solid product was dried under reduced pressure for 24 h; yield 80%. mPEG-*b*-PPC and PPC-*b*-PEG-*b*-PPC (samples 1–10): ^1^H-NMR (CD_3_CN-*d_3_*): δ (ppm) = 4.99–4.92 (CH PPC), 4.28–4.05 (CH_2_ PPC), 3.99–3.93 (CH_2_ PPC terminal unit), 3.74–3.36 (CH_2_ polyether), 3.29 (CH_3_-O), 3.01 (OH), 1.33–1.19 (CH_3_ PPC), 1.11–1.10 (CH_3_ PPC terminal unit). ^13^C-NMR (CD_3_CN-*d_3_*): δ (ppm) = 154.19 (C=O), 72.47 (CH), 70.18 (CH_2_ polyether), 68.88 (CH_2_ PPC), 15.36 (CH_3_).

### 2.5. General Procedure for Free-Radical Direct Miniemulsion Polymerization

For the PS nanoparticle synthesis 2 mg mPEG-*b*-PPC (Table 1, samples 2, 3, 4, 6) or 2 mg Lutensol AT50 were dissolved in 2 mL of water. A solution of styrene (56 µL), hexadecane (3.2 µL) and AIBN (0.5 mg) was added. The dispersion was stirred at 1000 rpm for 1 h. Subsequently, the dispersion was treated by ultrasonification at 70% amplitude for 2 min and further stirred for 24 h at 72 °C. The particles were washed by triple centrifugation at 6900 rpm and subsequent refilled with water. For IR measurements the emulsion was lyophilized. The solid content of the dispersions was around 1.6 wt %.

### 2.6. General Procedure for the Destabilization of the PS Nanoparticles

For the destabilization a stable PS emulsion (0.1 mL) was mixed with 1 ml concentrated hydrogen chloride. After 36 h the aggregated particles were collected by centrifugation at 4500 rpm and subsequently lyophilized.

### 2.7. Degradation Study of the Surfactant

For the online degradation study in a NMR tube mPEG_113_-*b*-PPC_49_ (17 mg) (Table 1, sample 3) was dissolved in 50 µL (7 vol %) CD_3_CN and mixed with 700 µL DCl/D_2_O (33% and pH 1). For the degradation study based on SEC measurements, mPEG_113_-*b*-PPC_49_ (50 mg) was dissolved in 200 µL (2 vol %) CH_3_CN and was mixed with 10 mL HCl/H_2_O (33% and pH 1). The samples were stirred for the whole reaction time. For each measurement an aliquot of 1 mL was taken, und the solvent was removed under reduced pressure.

## 3. Results and Discussion

### 3.1. Synthesis and Characterization of Polycarbonate-Polyether Surfactants

**Scheme 1:**
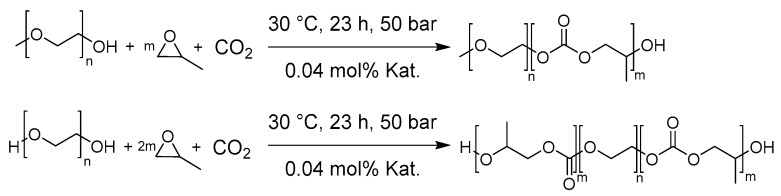
Synthetic scheme for mPEG-*b*-PPC und PPC-*b*-PEG-*b*-PPC surfactants based on mPEG and PEG as initiators, PO and CO_2_.

Amphiphilic di- and triblock copolymers were prepared by immortal polymerization of poly(ethylene glycol) monomethyl ether (mPEG) and poly(ethylene glycol) (PEG) with carbon dioxide and propylene oxide (PO) (Scheme 1). The synthesis protocol using (*R*,*R*)-(salcy)-Co(III)OBzF_5_ and bis(triphenylphosphine)iminium chloride ([PPN]Cl) as catalyst system was developed by our group in a previous work [26]. In the current work, it was varied by using mPEG initiators with more repeating units. This catalyst system was chosen, because it results in well-defined polycarbonates with a perfectly alternating incorporation of carbon dioxide and epoxide as well as very low molecular weight distribution [26,34]. It should be emphasized that the crude polymer was purified by precipitation in cold diethyl ether and by column chromatography using neutral aluminum oxide for complete removal of the catalyst.

**Figure 1:**
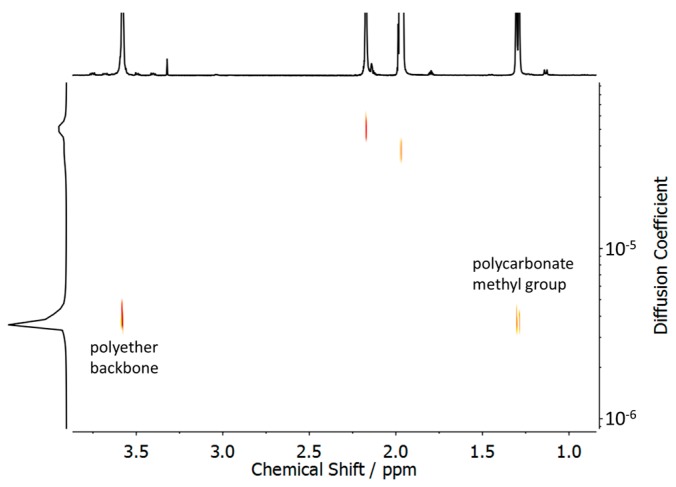
^1^H*-*DOSY-NMR spectrum (CD_3_CN, 400 MHz) of mPEG_113_-*b*-PPC_49_ (Table 1, sample 3).

Based on the degree of polymerization of a given mPEG or PEG the degree of polymerization was varied by adjusting the ratio between monomer and PEG initiator (Table 1). The characterization using ^1^H- and ^13^C-NMR spectroscopy and SEC corresponds to the work of Hilf et al. [26] and is shown in the Supporting Information (Figures S1–S5). Di- and triblock surfactants were synthesized with a narrow size distribution (*Đ* of 1.03 to 1.09) and adjustable molecular weights between 2800 g·mol^−1^ and 10,000 g·mol^−1^. The molecular weights determined by SEC are lower than the ^1^H-NMR values because of the hydrophobic character of the aliphatic polycarbonates and the resulting lower hydrodynamic radius. Furthermore, SEC was calibrated with a PEG standard. AB and ABA type polycarbonate surfactants based on mPEG_113_, mPEG_45_ and PEG_45_ were accessible. The successful synthesis of block copolymers without the formation of polycarbonate homopolymer was confirmed by ^1^H-DOSY-NMR spectroscopy (Figure 1). The polyether signal at 3.55 ppm and the methyl groups of the poly(propylene oxide) at 1.33–1.19 ppm show the same diffusion coefficient, supporting the formation of a block copolymer.

The critical micelle concentration (CMC) of the amphiphilic copolymers shows low values between 1 and 9 mg·L^−1^ [35,36], because of the highly hydrophobic character of the polycarbonates (Table 1 and Figure S6) [37]. Samples 8–10 are not soluble in water, so they cannot be used as surfactants for an oil-in-water emulsion. The comparison of the solubility of sample 8 and 3 indicates the influence of the block length on the aqueous solubility. The water-soluble sample 3 contains a large hydrophilic block and a smaller hydrophobic block, whereas the insoluble sample 8 is composed of two blocks with the same degree of polymerization. Besides the CMC the surface-active properties were also characterized by their hydrophilic-lipophilic-balance (HLB) values. The values were calculated by the method of Griffin [38]. The formal HLB values are between 16.6 and 5.6 and were calculated by the following equation (HLB = 20·(1 − *M*_1_/*M*)) with *M*_1_ the molar mass of the hydrophobic part and *M* the molar mass of the whole polymer). However, the HLB values are merely a first indication for the properties, because the hydrophilicity and hydrophobicity of the molecules are not included for the HLB calculation.

### 3.2. Surfactant Properties and Degradation

**Figure 2:**
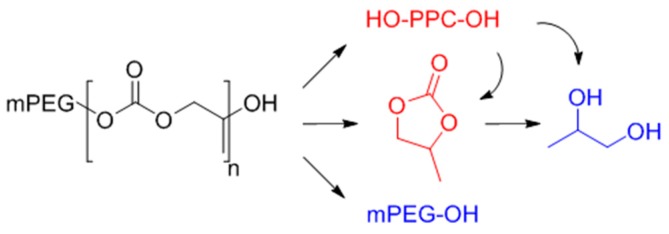
Degradation scheme for the acidic degradation of mPEG-*b*-PPC via hydrolysis (top) and depolymerization (middle). Hydrophobic (red) and hydrophilic (blue) degradation products are highlighted.

To investigate the degradation of the surfactants based on poly(ethylene glycol) and poly(propylene carbonate) in a poly(styrene) nanoparticle dispersion, the degradation of the pure block copolymer was characterized first. To the best of our knowledge, this is the first time that water-soluble polymers containing aliphatic polycarbonates based on carbon dioxide were investigated regarding their degradation in an acidic aqueous solution. Aliphatic polycarbonates can be degraded in different ways. They can be cleaved by a nucleophilic molecule and may depolymerize via backbiting of the terminal hydroxyl group. In Figure 2 the degradation products after incubation in an acidic aqueous solution are shown. The hydrophobic molecules are highlighted in red and the hydrophilic structures in blue. Cyclic carbonates, whose formation is thermodynamically favored [23,39], result from depolymerization via backbiting and can be hydrolyzed by water to diols. The hydrolysis of the backbone yields dihydroxyl-functionalized oligocarbonates and if this occurs at the block link, water-soluble poly(ethylene glycol). At first, the reaction solution is clear with a stable foam. During the reaction the foam disappears and the mixture becomes turbid (precipitated polycarbonate). After complete degradation the solution becomes clear again (Figure S7).

Because the amphiphilic polymer is not molecularly dissolved but rather in an aggregated state above the CMC, the degradation conditions have to be harsher than expected. It cannot be assumed that the hydrophobic cores of the aggregates are drained by the aqueous solution. Using an aqueous solution with a pD value as low as 1, the degradation was very slow at both room temperature (RT) and 50 °C, respectively. The degradation was monitored by ^1^H-NMR and SEC measurements. The measurement error for NMR spectroscopy is ±1%. After 51 days solely 3% (RT) or rather 11% (50 °C) polycarbonate units were degraded (Figure S8). At a reaction temperature of 70 °C the degradation is faster (86% after 51 days), because 70 °C is significantly higher than the glass transition temperature of the core [16]. However, this degradation rate is still not satisfactory for a rapid purification of the poly(styrene) dispersion. The SEC curves of each degradation condition show a broadening and a shift to higher elution volumes indicating chain scission (Figures S9–S11).

**Figure 3:**
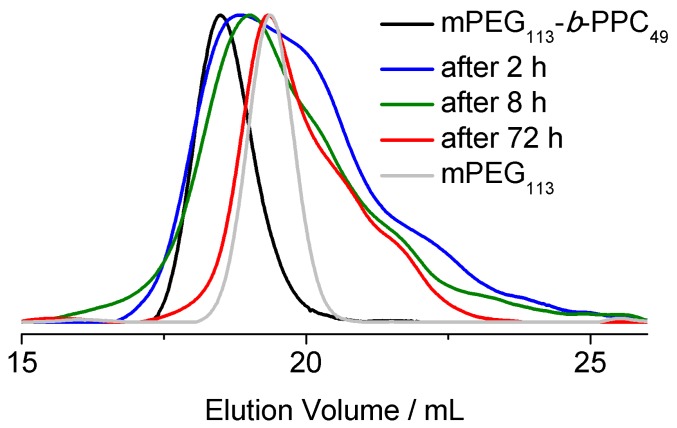
SEC results of the degradation study of mPEG_113_-*b*-PPC_49_ (Table 1, sample 3) in conc. hydrogen chloride in comparison with the mPEG_113_ initiator using DMF as eluent and PEG calibration.

To increase the degradation rate at room temperature, concentrated hydrochloric acid was employed. In Figure 3 the SEC results of collected samples during the degradation reaction of mPEG_113_-*b*-PPC_49_ (Table 1, sample 3) in comparison to the mPEG_113_ initiator are shown. After 2 h (blue) the size distribution is much broader than for the original surfactant. The peak maximum shifts to higher elution volumes and a multimodal distribution results. The hydrolysis decreases the molecular weight and results in polycarbonate homopolymers with unspecific molecular weights. After 8 h (green) of reaction time the peak maximum shifts to even higher elution volumes. After 72 h (red) the peak maximum reaches the elution volume of the non-degradable initiator and the size distribution becomes narrow again. A rather small amount of polycarbonate homopolymer still exists.

**Figure 4:**
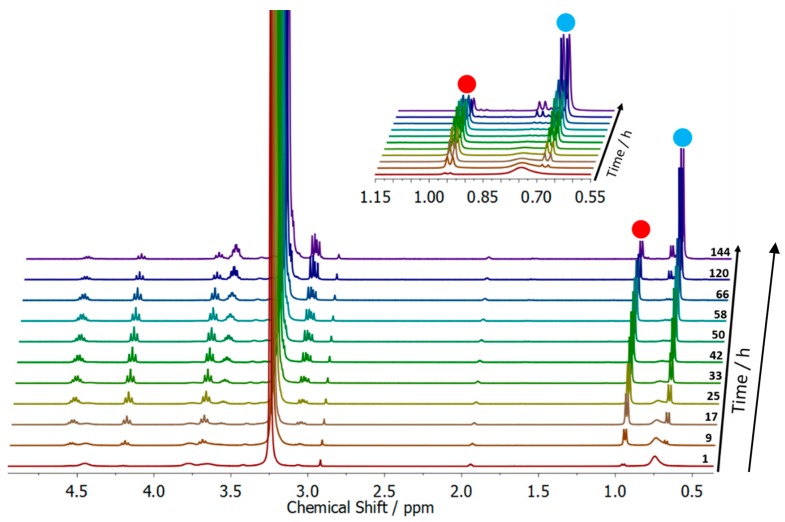
Selected spectra of the online ^1^H-NMR degradation kinetics of mPEG_113_-*b*-PPC_49_ (Table 1, sample 3) in conc. DCl (400 MHz) Bottom: overlay of spectra of the online ^1^H-NMR kinetic study. Top: zoom in, showing the increase and decrease of the methyl signals of propylene carbonate (red dot) and propane-1,2-diol (blue dot).

For a more precise investigation of degradation kinetics online ^1^H-NMR measurements were applied (Figure 4). To analyze the amount of degradation products (hydrolysis and depolymerization) the polyether backbone (3.29 ppm) was chosen as a constant integral with a known number of protons. The amount of propylene carbonate (depolymerization) and propane-1,2-diol (hydrolysis) was determinable by their methyl signals. The methyl signal of propylene carbonate occurs at 0.94 ppm (red dot) and the methyl signal of propane-1,2-diol at 0.68 ppm (blue dot). The signal of propylene carbonate increases faster than the signal of the diol, but after 38 h its value decreases. The increase and decrease of the signals can be translated to units of the degradation products per mPEG. Because of the surfactant’s aggregation in an aqueous solution, the signals of the water insoluble polycarbonates (0.74 ppm) are just visible fractionally and do not correspond to the values of measurements in CD_3_CN (Figure S1).

**Figure 5:**
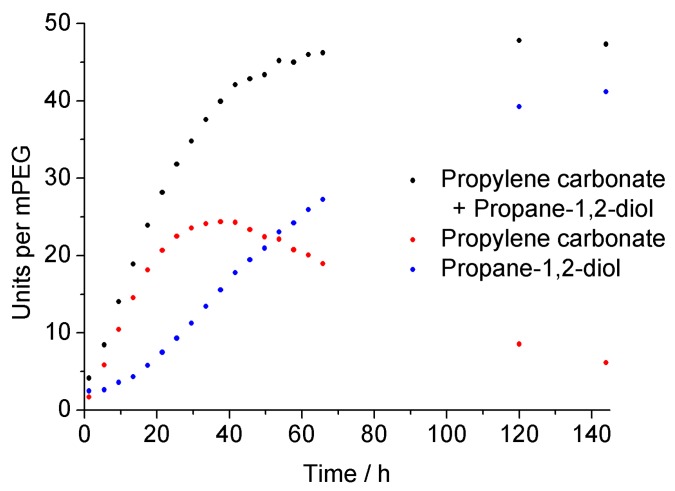
In situ ^1^H-NMR degradation study of mPEG_113_-*b*-PPC_49_ (Table 1, sample 3) in conc. DCl. Comparison of the degradation products propylene carbonate (red), propane-1,2-diol (blue) and both together (black) in units per mPEG_113_.

The degradation products per mPEG are plotted individually and together versus the time in Figure 5. The calculated amount of diols is too high at the beginning (10 h), due to signal overlap of the diols and the polycarbonates. Nevertheless, the propylene carbonate signals increase faster, but decrease later because of hydrolysis to propane-1,2-diol. There is an increase of propane-1,2-diol constant over the whole reaction time. Furthermore, the total amount of degradation products increased during the reaction and reached an amount of 95% after 3 days. The half-life (*T*_1/2_) is 18 h. These conditions (concentrated hydrochloric acid at room temperature) were chosen for the destabilization of the stable PS nanoparticle emulsions.

The acid-labile surfactants (sample 2, 3, 4 and 6) were investigated regarding their emulsifier properties in a free-radical miniemulsion polymerization of styrene droplets. Poly(styrene) nanoparticles were synthesized using the direct oil-in-water miniemulsion polymerization. Samples 8–10 were not soluble in water. None of the AB and ABA surfactants were soluble in cyclohexane, thus an inverse water-in-oil miniemulsion polymerization was not possible. All surfactants were used at a concentration of 1 mg·mL^−1^. Styrene, the osmotic pressure agent hexadecane, and the initiator AIBN were dispersed in an aqueous surfactant solution in water. In each case stable miniemulsions were obtained. This was expected for the samples 2–4 because of their HLB value exceeding 10, which suggests an effective surfactant for oil-in-water emulsions.

**Figure 6:**
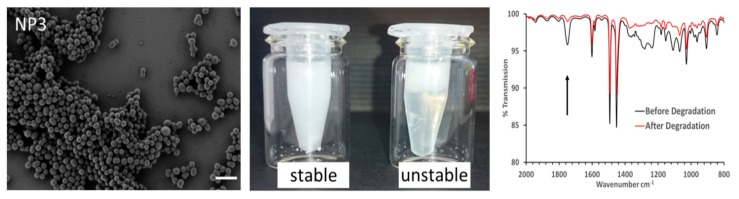
(**left**): SEM image of the synthesized PS nanoparticles (NP3). Scale bar = 1 µm. (**middle**): picture of a stable aqueous PS nanoparticle emulsion with mPEG_113_-*b*-PPC_49_ (Table 1, sample 3) as a surfactant and an instable emulsion after 36 h in conc. HCl. (**right**): FT-IR spectra of PS particles before (black) and after (red) degradation of the surfactant mPEG_113_-*b*-PPC_49_ (Table 1, sample 3).

A scanning electron microscopy (SEM) image of sample NP3 is shown in Figure 6, and the images of NP2, NP4 and NP6 are shown in the Supplementary Information (Figure S12). Furthermore, the nanoparticle diameter and PDI were determined by DLS (Table 2). The results show that each of the tested polycarbonate surfactants can be used to prepare stable polystyrene nanoparticles. Surfactants with HLB values above 10 stabilized PS nanoparticles efficiently with narrow size distribution (NP2, NP3 and NP4 in Table 2). The successful stabilization is independent of the length of the hydrophilic block (NP2 and NP4) and of the wt % of PPC (NP2 and NP3). Remarkably, the results of sample 3 (NP3) are comparable to established surfactants (Lutensol AT50) for this polymerization technique. However, if the hydrophobic block becomes too dominant and the HLB value decreases below 10, the surfactant produced PS dispersions with polydisperse size distribution, such as for sample NP6. Obviously, these amphiphilic systems are not suitable for direct miniemulsion polymerization with defined particle size distribution. Subsequent to the polymerization concentrated hydrochloric acid was added to the dispersion to degrade the surfactant and to precipitate the nanoparticles. Destabilization of the dispersion is desired, because isolation of the nanoparticles is simplified at the top of the aqueous solution (Figure 6).

Successful degradation and separation of the surfactants from polystyrene particles was proven by FT-IR (Figure 6). The FT-IR spectra of stable washed and destabilized PS nanoparticles are shown. The disappearance of the carbonyl stretch vibration of the carbonate groups at 1749 cm^−1^ supports a successful separation of particles and surfactants. The reference sample using Lutensol AT50 as a surfactant remained stable under these conditions (Figure S13).

## 4. Conclusions

Novel degradable AB and ABA polycarbonate-polyether block copolymers based on hydrophilic polyethers and hydrophobic aliphatic polycarbonates were synthesized by catalytic polymerization. mPEG and PEG of different molecular weights were used as macroinitiators for the direct polymerization of carbon dioxide and propylene oxide to create hydrophobic blocks based on aliphatic polycarbonates. A series of amphiphilic polymers was prepared with molecular weights of 2800 to 10,000 g·mol^−1^ with excellent *Đ* between 1.03 and 1.09. The amphiphilic properties were characterized by determining the CMC and calculating the HLB values using the method of Griffin. These surfactants were investigated with respect to their hydrolysis and depolymerization in acidic aqueous solution. Unexpectedly, enormous stability of the polycarbonate block under these conditions observed. Due to the high stability at pH 1 for 51 days with a degradation of 3% at room temperature and 11% at 50 °C, the degradation studies were additionally carried out in concentrated HCl for 144 h. Furthermore, their properties as surfactants were analyzed in a direct oil-in-water miniemulsion polymerization to generate stable PS nanoparticles. Depending on the block length and their ratio, nanoparticle sizes between 270 nm and 940 nm were prepared, with the new polycarbonate surfactants stabilizing the miniemulsion just as the highly established Lutensol AT50. Subsequent successful degradation and separation of the surfactants from the PS nanoparticles was proven by FT-IR.

The presented surfactants are obtained from very simple and easily accessible components in a solvent-free one-pot synthesis and enable direct use of carbon dioxide. Ideal conditions for commercially available products. The simplified purification of nanoparticles, using degradation of the surfactant under acidic conditions, shows their potential for the synthesis of surfactant free nanocarriers and acid-labile surfaces.

## Figures and Tables

**Table 1:** Overview of all characterization data for all AB and ABA copolymers.

Sample	Composition ^a^	*M_n_*/g·mol^−1 b^	*Đ* ^b^	*M_n_*/g·mol^−1 a^	CMC/mg·L^−1 c^	HLB ^d^
1	mPEG_113_-*b*-PPC_10_	5000	1.05	6000	2	16.6
2	mPEG_113_-*b*-PPC_20_	5300	1.08	7000	1	14.2
3	mPEG_113_-*b*-PPC_49_	6500	1.09	10,000	2	10.0
4	mPEG_45_-*b*-PPC_8_	2500	1.04	2800	9	14.2
5	mPEG_45_-*b*-PPC_10_	2600	1.03	3000	3	13.2
6	mPEG_45_-*b*-PPC_26_	3600	1.04	4600	3	8.5
7	mPEG_45_-*b*-PPC_35_	3900	1.08	5600	14	7.1
8	mPEG_45_-*b*-PPC_47_	4400	1.07	6800	n.s. ^e^	5.8
9	PPC_10_-*b*-PEG_45_-*b*-PPC_10_	3300	1.06	4000	n.s. ^e^	9.9
10	PPC_25_-*b*-PEG_45_-*b*-PPC_25_	4700	1.08	7100	n.s. ^e^	5.6

^a^ Terminology: Indices represent the absolute number of the respective repeating unit (rounded to integer), determined by ^1^H NMR spectroscopy, ^b^ determined by SEC in DMF calibrated with a PEG standard, ^c^ determined with a Du Nouy tensiometer, ^d^ calculated by the method of Griffin, ^e^ insoluble in aqueous solution.

**Table 2:** Overview of all synthesized PS nanoparticles and the surfactants applied for miniemulsion polymerization.

Sample ^a^	Surfactant	wt % PPC of Surfactant ^c^	Particle Size *d*/nm ^d^	PDI ^d^
NP2	2	29	330	0.12
NP3	3	50	270	0.08
NP4	4	29	940	0.08
NP6	6	57	140–>1000	0.57
NPL	Lut. AT50 ^b^	0	160	0.06

^a^ The label corresponds to the surfactant number in Table 1, ^b^ HLB value of 18, CMC = 25 mg·L^−1^ [40], ^c^ Terminology: Indices represent the absolute number of the respective repeating unit (rounded to integer), determined by ^1^H-NMR spectroscopy, ^d^ determined by dynamic light scattering (DLS).

## Supplementary Materials

The following are available online at www.mdpi.com/2073-4360/9/9/422/s1, Figure S1: ^1^H NMR spectrum of mPEG_113_-*b*-PPC_49_ (Table 1, sample 3) (400 MHz, CD_3_CN), Figure S2: ^13^C NMR spectrum of mPEG_113_-*b*-PPC_49_ (Table 1, sample 3) (100 MHz, CD_3_CN), Figure S3: SEC traces of all mPEG_113_-*b*-PPC AB-diblock copolymer surfactants (Table 1, sample 1–3) in comparison with the mPEG_113_ initiator using DMF as an eluent and PEG calibration, Figure S4: SEC traces of all mPEG_45_-*b*-PPC AB-diblock copolymer surfactants (Table 1, sample 4–8) in comparison with the mPEG_45_ initiator using DMF as an eluent and PEG calibration, Figure S5: SEC traces of all PPC-*b*-PEG_45_-*b*-PPC ABA-triblock copolymer surfactants (Table 1, sample 9,10) in comparison with the PEG_45_ initiator using DMF as an eluent and PEG calibration, Figure S6: Surface tension measurements of an aqueous solution of mPEG_113_-*b*-PPC_49_ (Table 1, sample 3) for the determination of the CMC, Figure S7: Degradation reaction of mPEG_113_-*b*-PPC_49_ (Table 1, sample 3) in aqueous hydrochloric solution (pH 1) after 8 days and 26 days at RT, 50 °C and 70 °C, respectively, Figure S8: Online ^1^H NMR degradation study of mPEG_113_-*b*-PPC_49_ (Table 1, sample 3) in hydrochloric aqueous solution (pD 1). Comparison of all degradation products in units per mPEG_113_ at different reaction temperatures (RT, 50 °C, 70 °C), Figure S9: SEC traces of the degradation study of mPEG_113_-*b*-PPC_49_ (Table 1, sample 3) in hydrochloric aqueous solution (pH 1) at room temperature in comparison with the mPEG_113_ initiator using DMF as an eluent and PEG calibration, Figure S10: SEC traces of the degradation study of mPEG_113_-*b*-PPC_49_ (Table 1, sample 3) in hydrochloric aqueous solution (pH 1) at 50 °C in comparison with the mPEG_113_ initiator using DMF as an eluent and PEG calibration, Figure S11: SEC traces of the degradation study of mPEG_113_-*b*-PPC_49_ (Table 1, sample 3) in hydrochloric aqueous solution (pH 1) at 70 °C in comparison with the mPEG_113_ initiator using DMF as an eluent and PEG calibration, Figure S12: SEM image of the synthesized PS nanoparticles (NP2, NP4 and NP6). Scale bar = 1 µm, Figure S13: Picture of a stable aqueous nanoparticle dispersion with LutAT50 as a surfactant before (left) and after (right) the treatment with conc. HCl solution (36 h).
