# Supplementary material for: Acid-Labile Surfactants Based on Poly(ethylene glycol), Carbon Dioxide and Propylene Oxide: Miniemulsion Polymerization and Degradation Studies
Source: Polymers (Basel). 2017 Sep 6;9(9):422. doi: 10.3390/polym9090422 (PMC6419001; doi:10.3390/polym9090422)
Supplement: Supplementary file 1 [file polymers-09-00422-s001.pdf]

Supplementary Materials: Acid-Labile Surfactants Based on Poly(ethylene glycol), Carbon Dioxide and Propylene Oxide: Miniemulsion Polymerization and Degradation Studies

Markus Scharfenberg, Sarah Wald, Frederik R. Wurm and Holger Frey

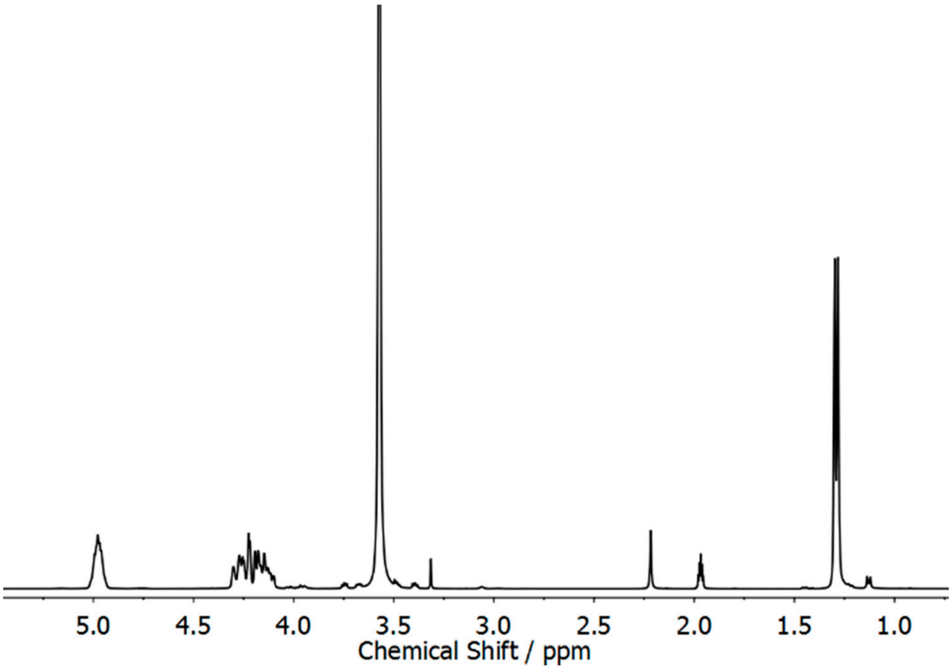

Figure S1. ^{1}H NMR spectrum of mPEG$_{113}$-*b*-PPC$_{49}$ (Table 1, sample 3) (400 MHz, CD_3CN).

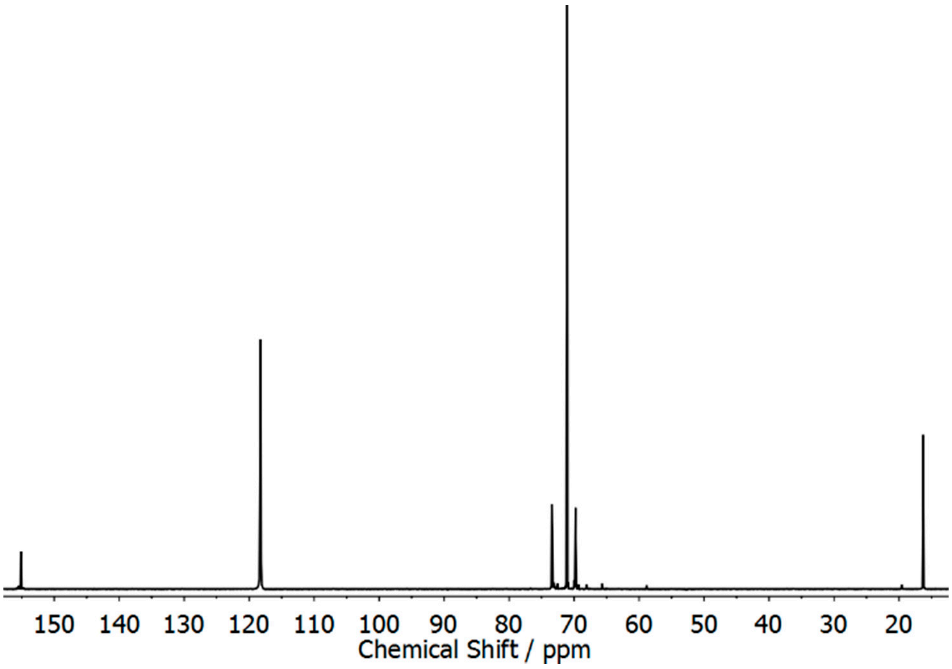

Figure S2. ^{13}C NMR spectrum of mPEG$_{113}$-*b*-PPC$_{49}$ (Table 1, sample 3) (100 MHz, CD_3CN).

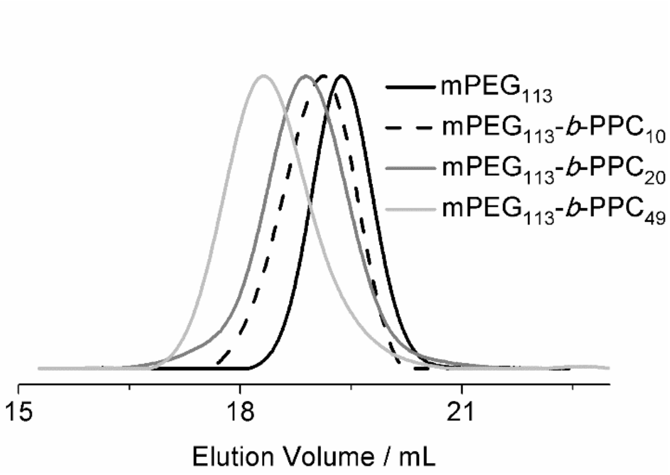

Figure S3. SEC traces of all $mPEG_{113}$-*b*-PPC AB-diblock copolymer surfactants (Table 1, sample 1–3) in comparison with the $mPEG_{113}$ initiator using DMF as an eluent and PEG calibration.

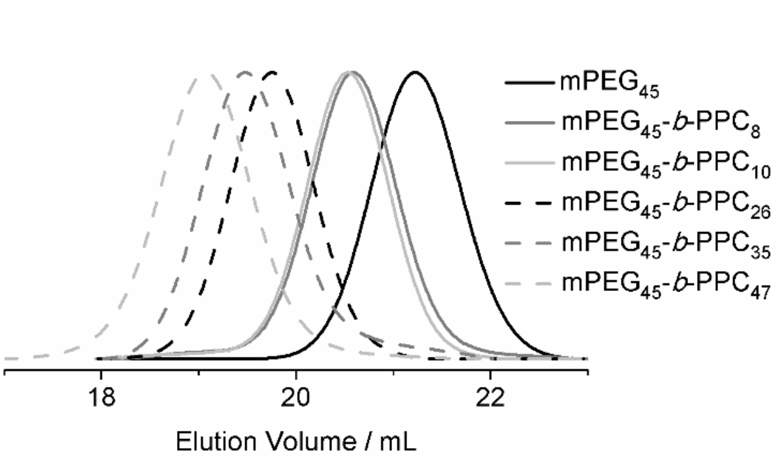

Figure S4. SEC traces of all $mPEG_{45}$-*b*-PPC AB-diblock copolymer surfactants (Table 1, sample 4–8) in comparison with the $mPEG_{45}$ initiator using DMF as an eluent and PEG calibration.

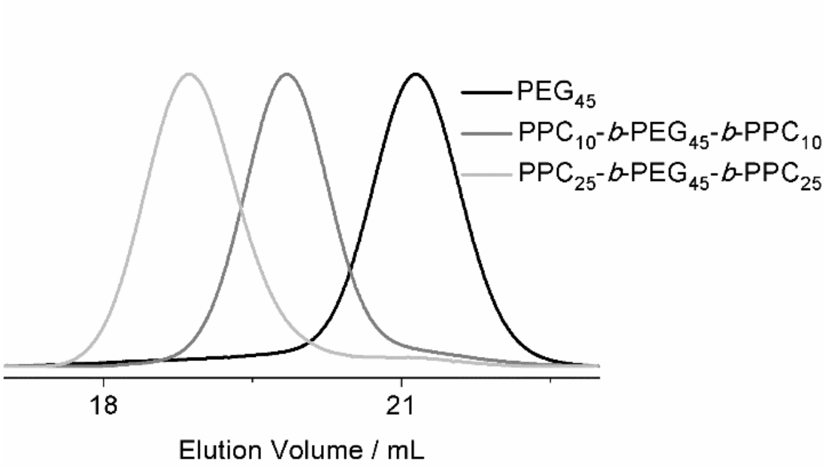

Figure S5. SEC traces of all PPC-*b*-PEG_{45}-*b*-PPC ABA-triblock copolymer surfactants (Table 1, sample 9,10) in comparison with the PEG_{45} initiator using DMF as an eluent and PEG calibration.

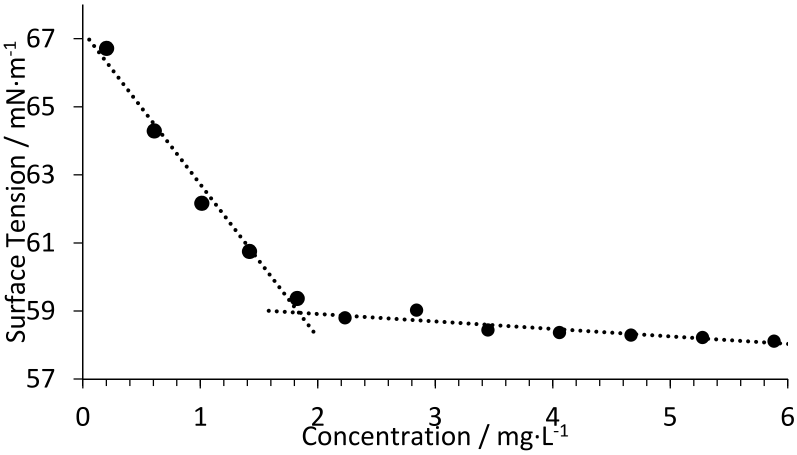

Figure S6. Surface tension measurements of an aqueous solution of $mPEG_{113}$-*b*-PPC_{49} (Table 1, sample 3) for the determination of the CMC.

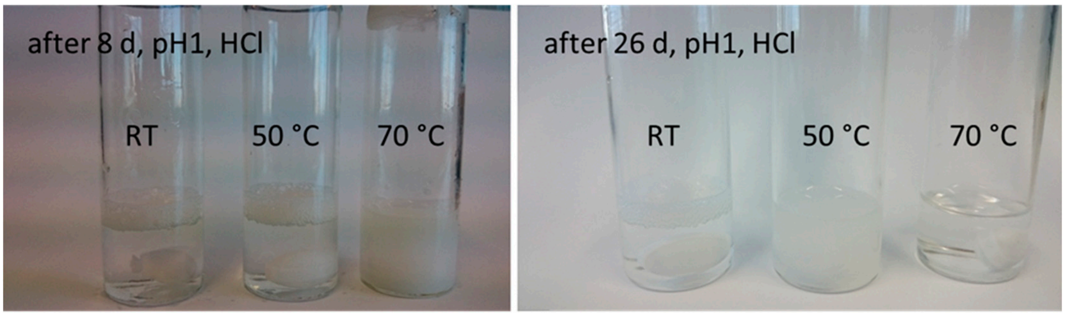

Figure S7. Degradation reaction of $mPEG_{113}$-*b*-PPC_{49} (Table 1, sample 3) in aqueous hydrochloric solution (pH 1) after 8 d and 26 d at RT, 50 °C and 70 °C, respectively.

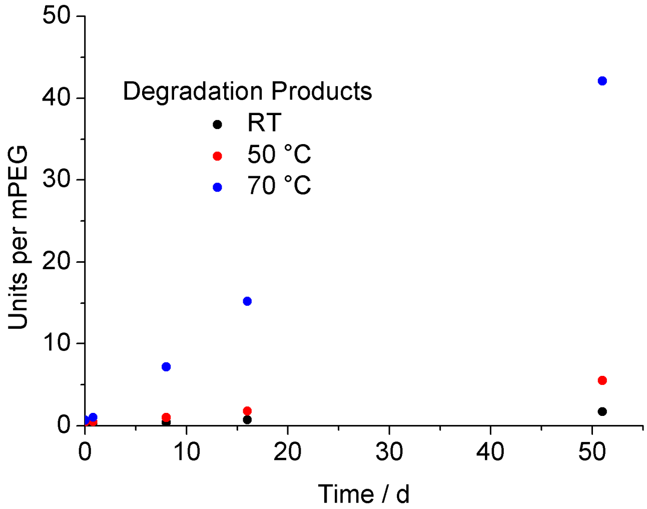

Figure S8. Online ^{1}H NMR degradation study of $mPEG_{113}$-*b*-PPC_{49} (Table 1, sample 3) in hydrochloric aqueous solution (pD 1). Comparison of all degradation products in units per $mPEG_{113}$ at different reaction temperatures (RT, 50 °C, 70 °C).

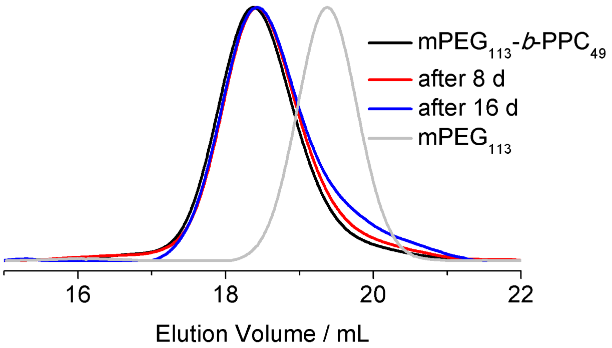

Figure S9. SEC traces of the degradation study of mPEG113-*b*-PPC49 (Table 1, sample 3) in hydrochloric aqueous solution (pH 1) at room temperature in comparison with the mPEG113 initiator using DMF as an eluent and PEG calibration.

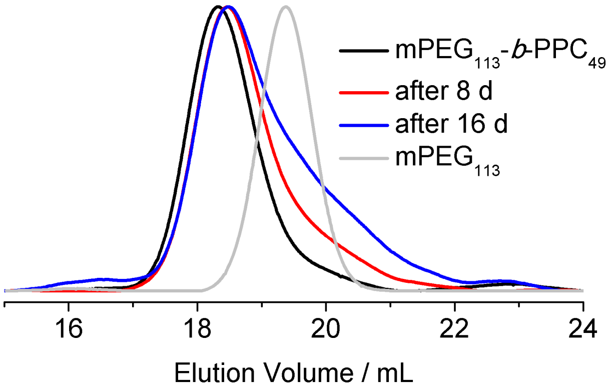

Figure S10. SEC traces of the degradation study of mPEG113-*b*-PPC49 (Table 1, sample 3) in hydrochloric aqueous solution (pH 1) at 50 °C in comparison with the mPEG113 initiator using DMF as an eluent and PEG calibration.

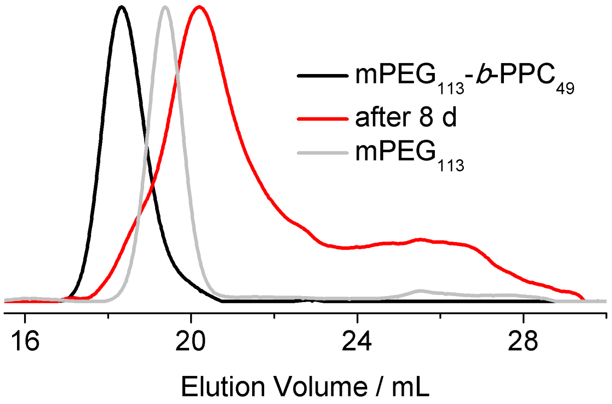

Figure S11. SEC traces of the degradation study of mPEG113-*b*-PPC49 (Table 1, sample 3) in hydrochloric aqueous solution (pH 1) at 70 °C in comparison with the mPEG113 initiator using DMF as an eluent and PEG calibration.

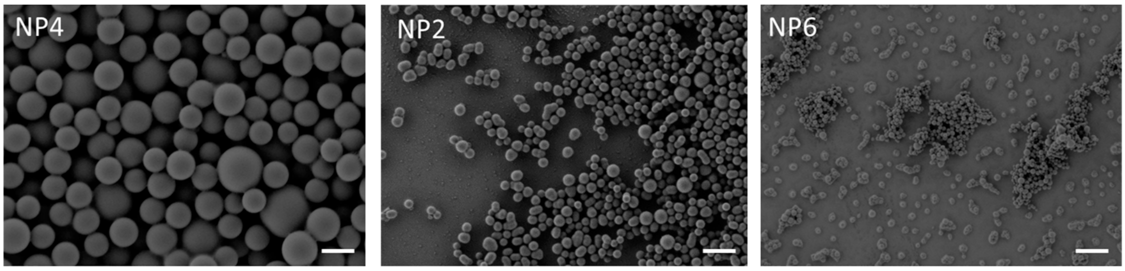

Figure S12. SEM image of the synthesized PS nanoparticles (NP2, NP4 and NP6). Scale bar = 1 μm.

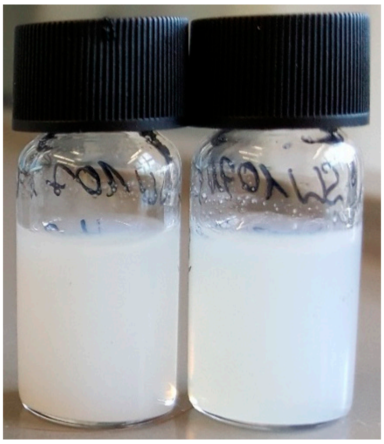

Figure S13. Picture of a stable aqueous nanoparticle dispersion with LutAT50 as a surfactant before (left) and after (right) the treatment with conc. HCl solution (36 h).
